# An Immune-Related lncRNA Pairing Model for Predicting the Prognosis and Immune-Infiltrating Cell Condition in Human Ovarian Cancer

**DOI:** 10.1155/2022/3168408

**Published:** 2022-08-16

**Authors:** Xiaocui Zhang, Qing Yang

**Affiliations:** Department of Obstetrics and Gynecology, Shengjing Hospital of China Medical University, Shenyang, Liaoning 110004, China

## Abstract

Ovarian cancer is the second common cancer among the gynecological tumors. It is difficult to be found and diagnosed in the early stage and easy to relapse due to chemoresistance and deficiency in choices of treatment. Therefore, future exploring the biomarkers for diagnosis, treatment, and prognosis prediction of ovarian cancer is significant to women in the world. We downloaded data from TCGA and GTEx and used R “limma” package for analyzing the differentially expressed immune-related lncRNA in ovarian cancer and finally got 7 downregulated and 171 upregulated lncRNA. Then, we paired the differentially expressed immune-related lncRNA and constructed a novel lncRNA pairing model containing 7 lncRNA pairs. Based on the cut-off point with the highest AUC value, 102 patients were selected in high-risk group and 272 in low-risk group. The KM analysis suggested that the patients in the low-risk group had a longer overall survival. Future analysis showed the correlations between risk scores and clinicopathological parameters and infiltrating immune cells. In conclusion, we identified an immune-related lncRNA pairing model for predicting the prognosis and immune-infiltrating cell condition in human ovarian cancer, which thus further can instruct immunotherapy.

## 1. Introduction

As reported, there were 313,959 cases of newly diagnosed ovarian cancer (OC) and 207,252 new deaths for OC in 2020 [1]. OC is the second common cancer to cause large death among the gynecological tumors, difficult to be found and diagnosed in the early stage of disease, deficient in choices of treatment, and easy to relapse [2–5]. Therefore, future exploring the biomarkers to diagnose, treat, and predict the prognosis of OC is of significance to women.

Tumor immune-infiltrating cells are strongly correlated with cancer prognosis and response to therapy. Ye et al. reported that tumor immune-infiltrating cells, especially neutrophils, Tregs, and macrophages, affected the clinical outcome in patients with colorectal cancers and could be markers to predict the prognosis and response to therapy [6]. Qi et al. also reported that the accumulation of CD39 + CD8+ T cells in tumor microenvironment indicated poor prognosis in clear cell renal cell carcinoma and benefit of tyrosine kinase inhibitors therapy [7]. And different methods to target different immune-infiltrating cells, such as lymphocytes [8, 9], dendritic cells [10], and nature killer cells [11], in the TME have been a popular and effective therapy.

As we know, long noncoding RNA (lncRNA) is a noncoding RNA with a length of more than 200 nucleotides. Studies have shown that lncRNA plays an important role in many life activities, such as dose compensation effect, epigenetic regulation, cell cycle regulation, and cell differentiation regulation, and has become a hot spot in genetic research [12]. For example, lncRNA MALAT1 plays an antiapoptotic and anti-inflammatory role in the brain microvascular system to reduce ischemic cerebrovascular and parenchymal damage, which can be a therapeutic target to minimize brain damage after stroke [13]. A recent study reported that lncRNA H19X could regulated the expression of TGF-*β*, regulating differentiation and survival of myofibrillar cells [14]. Besides, lncRNA, such as MEG3 and Kcnq1ot1, can regulate the progress of heart disease [15, 16]. More importantly, lncRNA is included in the prediction of cancer prognosis [17–24] and can be a regulatory factor of tumor immune microenvironment including immune-infiltrating cells and immune cell function [25, 26]. Previous study had emphasized the importance of lncRNA in OC, among which lncRNA facilitates epithelial-mesenchymal transition (EMT) and invasion-metastasis and other tumor behaviors in OC [27–30]. In addition, there were many reports saying tumor immune infiltration-related lncRNA model to predict prognosis and instruct immunotherapy of patients in non-small-cell lung cancer [22, 31], bladder cancer [20, 32, 33], liver hepatocellular carcinoma [23, 24, 34], breast cancer [35–37], colon cancer [38], glioma [21, 39, 40], and so on [41–43]. Therefore, it is very helpful to perform combined analysis of lncRNA and immune-infiltrating cells for OC diagnosis, treatment, and prognosis.

Here, we used a new algorithm to explore a new immune-related lncRNA pairing model for predicting the prognosis and immune-infiltrating cell condition in human OC based on The Cancer Genome Atlas (TCGA) and The Genotype-Tissue Expression (GTEx) database, which can help a lot in the improvement of prognosis prediction and immune therapy.

## 2. Materials and Methods

**Figure 1:**
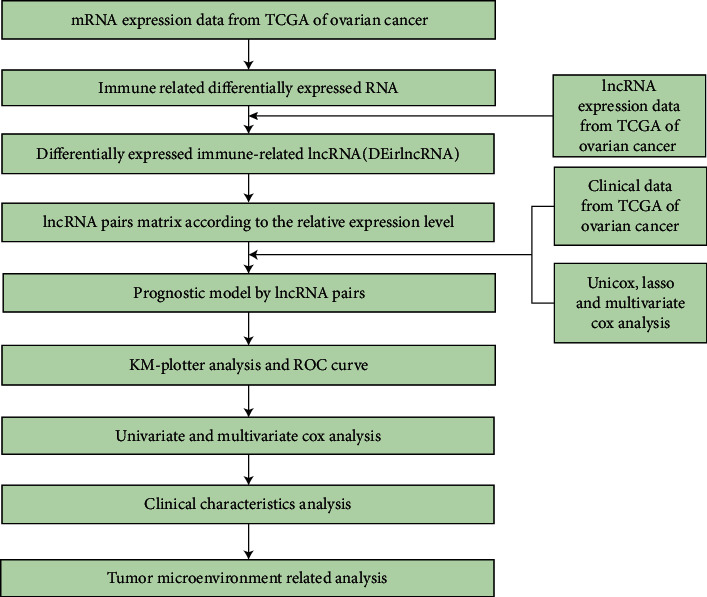
The flow chart of whole process of data analysis.

The data analysis steps are in Figure 1.

### 2.1. Data Preparation and Differentially Expressed Analysis

The RNA-seq data and clinical information of OC patients were gotten from https://portal.gdc.cancer.gov/repository. The RNA-seq data of patients having normal ovarian tissue were gotten from https://xenabrowser.net/datapages/. Data type was HTseq-FPKM, and gene expression level in both two databases was further processed by log2 (FPKM+1). The cases without clinical information and the repeated cases were removed. The data from Ensembl (http://asia.ensembl.org) were taken for RNA-type annotation. Genes in ImmPort database (http://www.immport.org) were taken for coexpression analysis of the differentially expressed immune-related lncRNA (gene correlation coefficients >0.4 and *p* value <0.001). Wilcoxon signed-rank test based on “limma” R package were used for differentially expressed immune-related lncRNA (DEirlncRNA) analysis in tumor and normal tissues (false discovery rate (FDR) <0.01).

### 2.2. DEirlncRNA Pairing

Considering the general applicability of the model and avoiding batch correction, lncRNA pairs matrix was constructed. One lncRNA pair had lncRNA 1 and lncRNA 2. If the expression value of lncRNA 2 is lower than lncRNA 1, consider the sample as 1; otherwise, the sample is defined as 0. Next, the matrix was further analyzed. If all the value of the lncRNA pairs in the samples were 0 or 1, the lncRNA pair was thought not be related to prognosis because there is no specific rank of pairing that cannot correctly predict the survival outcome of patients. When the proportion of expression proportion was 0 or 1 in one lncRNA pair exceeding 20% of total pairs, it was considered a significant pair.

### 2.3. Construction of the Prognostic Model by lncRNA Pairs

A prediction model of lncRNA pairs was constructed by univariate, lasso, and multivariate Cox regression analysis. The model was determined by ten-fold cross-validation and *p* value less than 0.01. The model with the highest point of area under curve (AUC) value was selected for further analysis. The risk score was calculated according to the standardized expression value of each pair and its corresponding coefficient. The formula was score = *e*^sum (expression of each gene pair × corresponding coefficient)^ . The receiver operating characteristic (ROC) curve was evaluated to determine the point of which the sum of sensitivity and specificity reached the highest, which was the cut-off point to divide patients into two groups.

### 2.4. Validation of the Prognostic Model of the lncRNA Pairs

The Kaplan-Meier (KM) analysis based on the “survive” and “survminer” packages showed the differences in survival time of the two groups. The KM analysis estimates the survival curve in this way: first, calculate the probability that patients who have lived for a certain period will live for the next period (i.e. survival probability), and then multiply the survival probability one by one, that is, the survival rate of the corresponding period. R software was also used to show the risk score values and survival status of each sample in the model. The “survivalROC” package was taken for predicting survival status in ROC curves.

### 2.5. Clinical Evaluation of the Prognostic Model by lncRNA Pairs

The chi-square test based on the “ComplexHeatmap” R package was taken for analyzing the relationship between the model and clinicopathological parameters (∗∗∗ means *p* < 0.001, ∗∗ means *p* < 0.01, and ∗ means *p* < 0.05). The risk scores of these clinicopathological features were compared between different groups by the Wilcoxon signed-rank test. The relationship between the risk score and clinicopathological parameters was performed by univariate and multivariate Cox regression analyses to demonstrate whether the model can be used to predict the prognosis independently. The “limma” and “ggupbr” packages were used to show the results in a format of forest maps.

### 2.6. Evaluation of Immune-Infiltrating Cells and Immune Checkpoint Genes Expression by the Prognostic Model of the lncRNA Pairs

Using several immune-related databases, the correlation between the risk score and immune cell condition was analyzed by Spearman correlation analysis (*p* value <0.05). The Wilcoxon signed-rank test based on the “ggplot2” packages was conducted for evaluating the content differences of immune-infiltrating cells between the two groups, and we showed the results in a box diagram. The “ggstatsplot” R package was performed to analyze the expression differences of immune checkpoint-related genes.

## 3. Results

### 3.1. DEirlncRNA Screening

**Figure 2:**
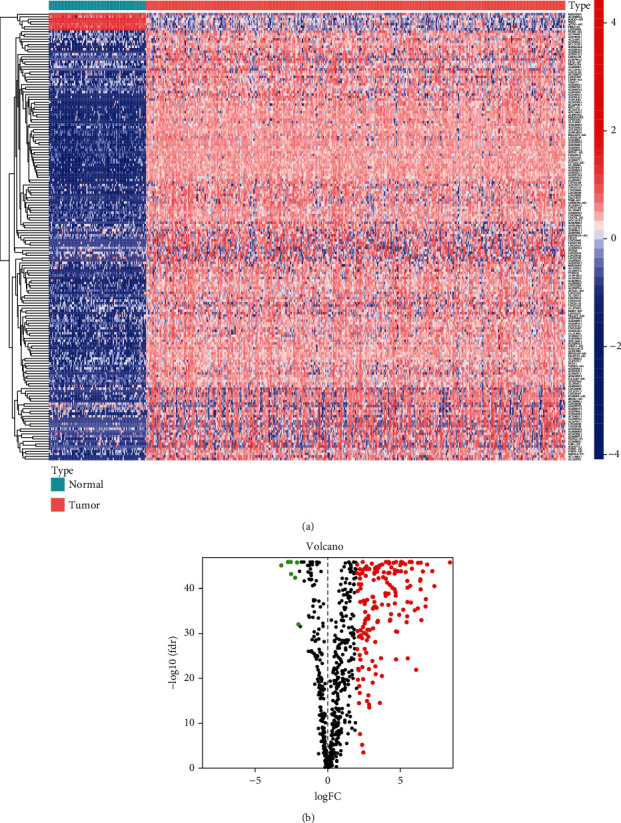
Screening the differentially expressed immune-related lncRNA (DEirlncRNA) in the TCGA datasets. The results were shown in the form of heatmap (a) (noted that the redder square in the heatmap meant the higher gene expression level, while the bluer ones meant the lower gene expression level) and volcano plot (b) (noted that the red dots in the volcano meant the upregulated genes, while the black ones meant the downregulated genes).

The expression data were gotten from TCGA database and GTEx database, which included 379 OC patients and 88 normal patients. Gene expression level in both two databases was further processed by log2 (FPKM+1). And we annotated the expression data with Ensembl GTF files, performed a correlation of immune-related gene and lncRNA, and got 694 immune-related lncRNA (Supplementary material 1). Compared the gene expression level of normal ovarian tissues in GTEx database and OC tissues in TCGA database, 178 differentially expressed immune-related lncRNA (Figure 2(a); Supplementary material 2) were identified, which included 7 downregulated and 171 upregulated (Figure 2(b)).

### 3.2. DEirlncRNA Pairing and Construction of the Prognostic Model by lncRNA Pairs

**Figure 3:**
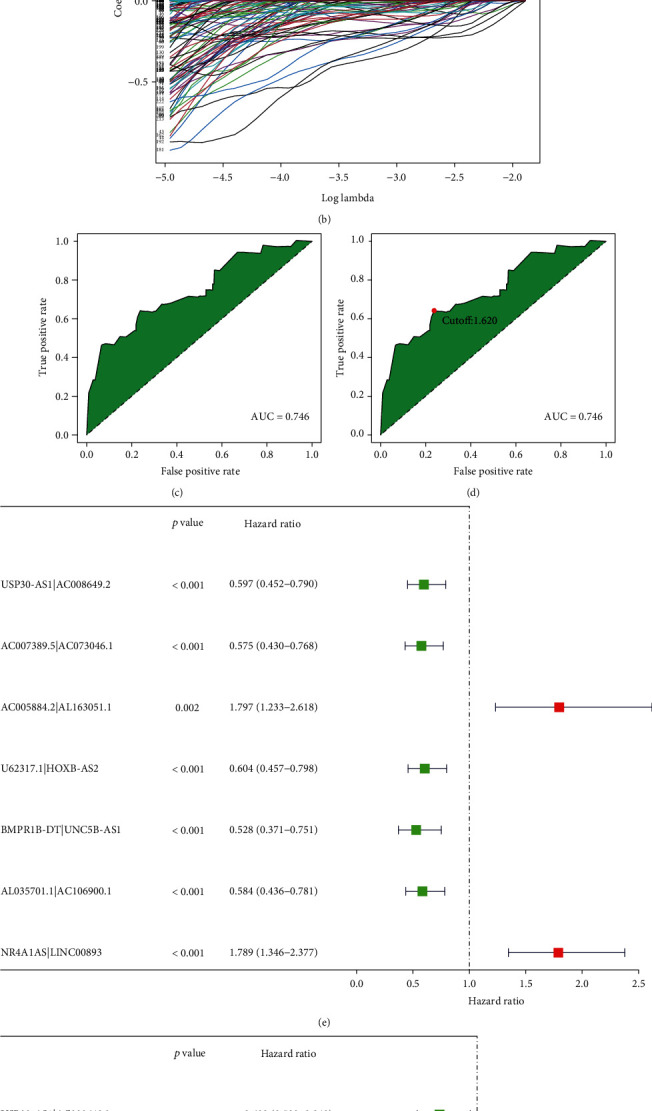
Construction of the prognostic model by lncRNA pairs. 7 immune-related lncRNA pairs were identified by a lasso regression analysis; (a) penalty term parameters to choose lambda value and (b) the relationship between lambda and regression coefficient. (c) The ROC of 7 immune-related lncRNA pairs had the maximum AUC (AUC = 0.746). (d) The ROC curve was evaluated to determine the point of which the sum of sensitivity and specificity reached the highest, which was considered as the cut-off point to distinguish between the high-risk and low-risk scores of OC patients (cut − off value = 1.620). Univariate (e) and multivariate (f) Cox analysis results of immune-related lncRNA pairs and survival time (*p* < 0.01 of each lncRNA pair included in the model).

After an iteration loop using lncRNA pairs matrix screening, we got 11984 DEirlncRNA pairs (Supplementary material 3). First, uniCox regression analysis was conducted, and 227 DEirlncRNA pairs were related to overall survival time (Supplementary material 4). Next, lasso Cox analysis suggested that 7 DEirlncRNA pairs had the highest point of AUC value (Figures 3(a)–3(c)).  Table 1 shows the coefficient, hazard ratio (HR), 95% confidence interval of HR, and *p* value of each lncRNA pair included in the model. Besides, we analyzed the AUC curve and found the point of which the sum of sensitivity and specificity reached the highest (Figure 3(d)), and we considered this value to be the cut-off point to divide the patients into two groups (high-risk and low-risk, Supplementary material 5). And we conducted univariate and multivariate Cox analyses, which indicated that 7 pairs were related to the overall survival (Figures 3(e) and 3(f)).

### 3.3. Validation of the Prognostic Model of the lncRNA Pairs

**Figure 4:**
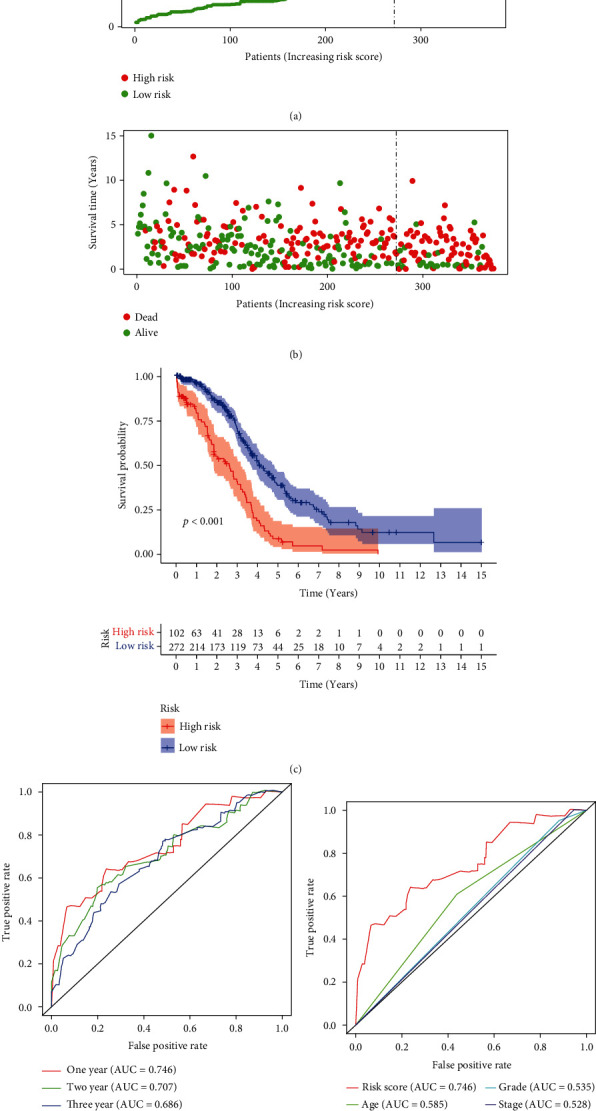
Validation of the prognostic model of the lncRNA pairs. Risk score values (a) and survival status (b) of OC patients. (c) KM plot of our model showed a good prognosis predicting potential (*p* < 0.001). (d) The ROC curve at 1, 2, and 3 years (AUC of 1 year at 0.746, AUC of 2 years at 0.707, and AUC of 3 years at 0.686). (e) The 1-year cliROC curve (AUC of risk scores at 0.746, AUC of age at 0.585, AUC of grade at 0.535, and AUC of stage at 0.528).

According to the cut-off point, we divided the patients into two groups, one containing 102 high-risk patients and the other containing 272 low-risk patients (Figure 4(a); Supplementary material 5). In addition, we draw the scatter figure to show the survival status and the risk score of each patient (Figure 4(b)). KM analysis showed that the patients with high-risk score survived shorter (*p* value <0.001, Figure 4(c)). The ROC curves at 1, 2, and 3 years indicated that all AUC were more than 0.7 (Figure 4(d)). What is more, the ROC curve at 1 year compared with other common clinicopathological parameters suggested that the model we developed had a perfect prediction ability (Figure 4(e)).

### 3.4. Clinical Evaluation of the Prognostic Model by lncRNA Pairs

**Figure 5:**
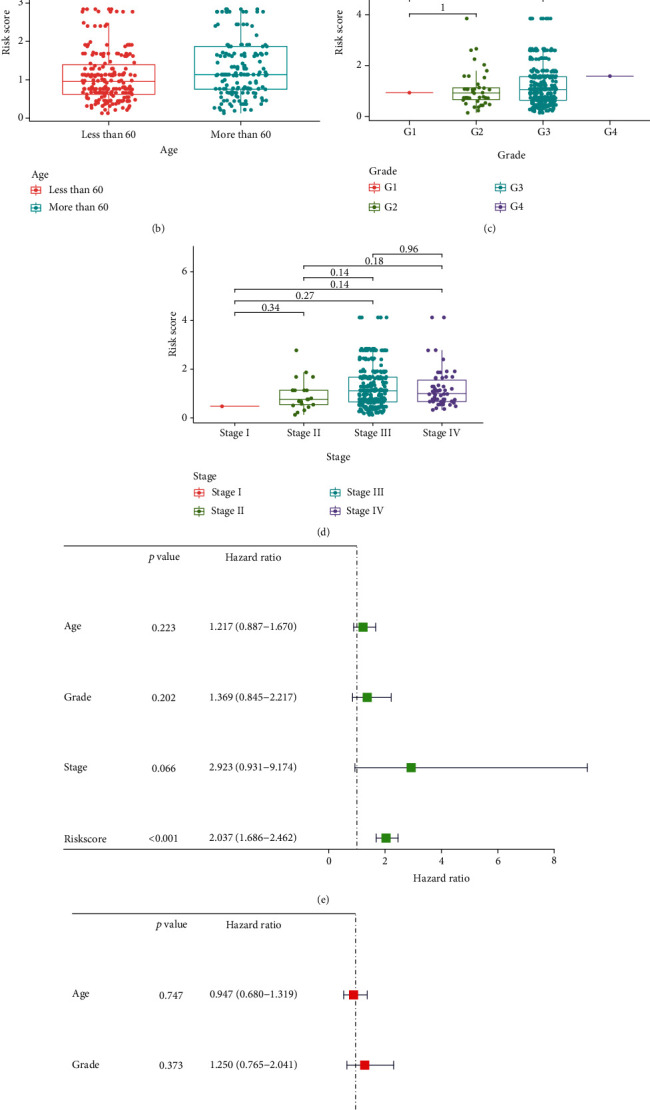
Clinical evaluation of the prognostic model by lncRNA pairs. (a) The chi-square test and (b–d) the Wilcoxon signed-rank test of risk score and clinicopathological parameters showed that age was significantly lower in the low-risk group (*p* = 0.0017), while grade and stage were higher in the high-risk group without significance. (e) UniCox and (f) multiCox analysis to predict the mode independence in prognosis (*p* < 0.001 of risk score calculated by the model).

The chi-square test suggested that age was significantly associated with the risk score (*p* < 0.001), but in the clinical stage, tumor grade had no differences between the two groups (Figure 5(a)). The risk scores of these clinicopathological features comparing between different groups by the Wilcoxon signed-rank test indicated that age had a positive correlation with the risk score (Figure 5(b)), while the other had no correlation (Figures 5(c) and 5(d)). The univariate (Figure 5(e)) and multivariate (Figure 5(f)) Cox regression analyses confirm that the model can be used to predict the prognosis independently.

### 3.5. Evaluation of Immune-Infiltrating Cells and Immune Checkpoint Genes Expression by the Prognostic Model of the lncRNA Pairs

**Figure 6:**
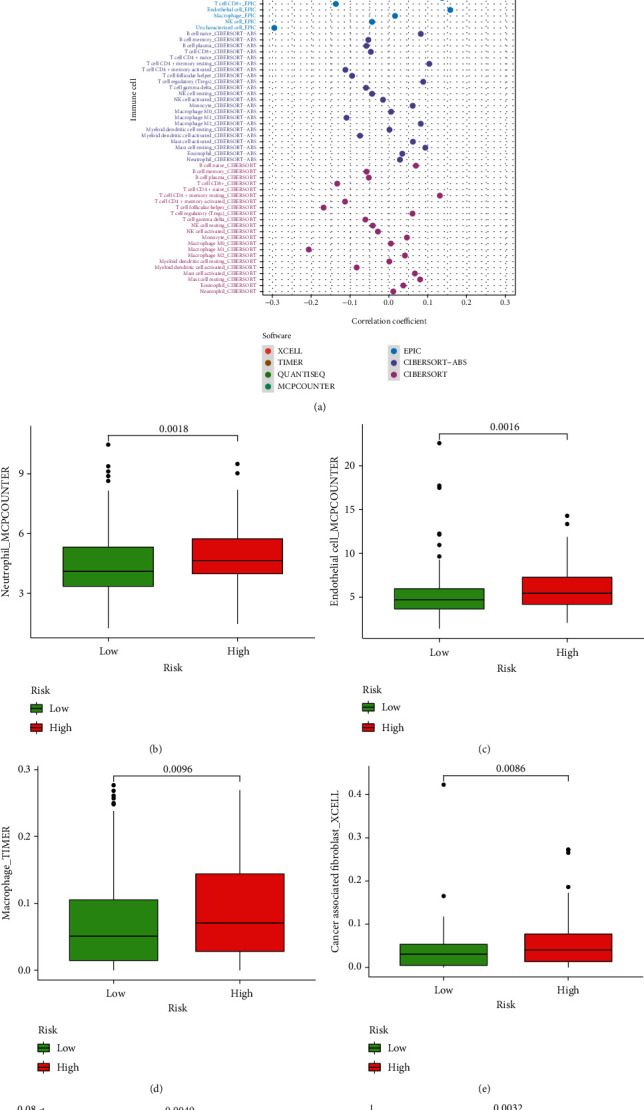
Evaluation of immune-infiltrating cells by the prognostic model of the lncRNA pairs. (a) Patients with different risk score had different immune cells infiltrating (*p* < 0.05). (b–g) Spearman correlation analysis of risk score and tumor-infiltrating immune cells showed high infiltration of neutrophil, endometrial cell, macrophage, cancer-associated fibroblast, T cells, and mast cells in the high-risk group (*p* < 0.01).

**Figure 7:**
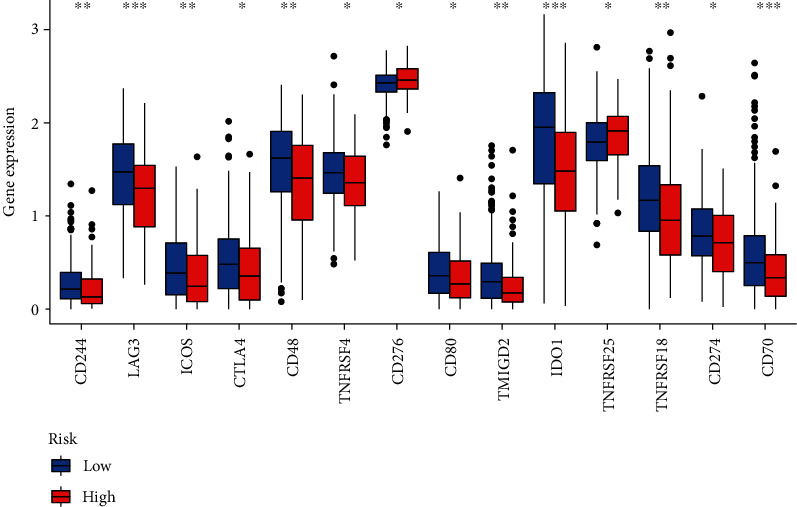
Evaluation of immune checkpoint genes expression by the prognostic model of the lncRNA pairs. CD244, LAG3, ICOS, CTLA4, CD48, TNFRSF4M, CD80, TMIGD2, IDO1, TNFRSF18, CD274, and CD40 were significantly lower in the high-risk group, while CD276 and TNFRSF25 were higher (∗∗∗ means *p* < 0.001, ∗∗ means *p* < 0.01, and ∗ means *p* < 0.05).

Through analysis based on several immune-related databases, we concluded that patients with different risk score had different immune cells infiltrating (Figure 6(a); Supplementary material 6). Through spearman analysis, we concluded that the high-risk scores were positively correlated with high infiltration of neutrophil, endometrial cell, macrophage, cancer-associated fibroblast, T cells, and mast cells (Figures 6(b)–6(g)). What is more, we evaluated the immune checkpoint genes expression levels between two groups, indicating that CD244, LAG3, ICOS, CTLA4, CD48, TNFRSF4M, CD80, TMIGD2, IDO1, TNFRSF18, CD274, and CD40 were significantly lower in the high-risk group, while CD276 and TNFRSF25 were higher (Figure 7; Supplementary material 7).

## 4. Discussion

Since OC causes so many deaths in the world every year, it deserves more in-depth exploration and research. TCGA and GTEx are platforms collecting clinical data, genomic variation, mRNA expression, miRNA expression, methylation, and other data of patients with or without human cancers, which is a very important data source for cancer researchers. Here, the clinical data and mRNA expression data of OC in TCGA database and the mRNA expression data of patients without OC in GTEx database were downloaded. First, we used coexpression analysis to get the differentially expressed immune-related lncRNA. Then, we constructed a novel model to predict the prognosis of OC based on lncRNA pairing. At the same time, we got the cut-off point to divide the patients into the high-risk and low-risk group. This model was verified by ROC curves and survival analysis, as well as the relationship between risk score and other clinicopathological parameters or immune-infiltrating cells. The results indicated that this model can instruct the prognosis prediction and immune therapy of OC.

There were many studies involving the role of lncRNA in OC, where lncRNA expression data was analyzed and lncRNA model was constructed to predict the prognosis of patients with OC [44–48]. Nevertheless, this model needed to measure the exact expression level of the lncRNA, which requires equipment with higher quality and expert to read the results. Besides, this model was verified by multivariate Cox analysis with a *p* value of 0.02. In our study, we used lncRNA pairing method in OC prognosis, with which we only needed to check the relative expression of the lncRNA pair instead of the exact expression level of each lncRNA. And we did the multivariate Cox analysis with a *p* value <0.001. It is reported that USP30-AS1 in our model also participated in the other immune-related lncRNA model based on TCGA and GTEx database, implying the importance of USP30-AS1 in OC [48]. Besides, several studies reported the role of USP30-AS1 in carcinogenesis [48–52]. But there were no studies of USP30-AS1 in OC, which needed to be future explored. lncRNA UNC5B-AS1 in our model had been reported to be an oncogenic gene in OC through regulating the H3K27me on NDRG2 via EZH2 [53]. Other lncRNA in our model had been reported in other cancer types, but no reports in OC. Therefore, we also need to do a lot of work, including not only prospectively collecting clinical samples to verify our model, but also studying the specific role of lncRNA involved in the model in ovarian cancer.

What is more, we first combined lncRNA with tumor immune-infiltrating cell condition in OC. We found that the proportion of neutrophil, endometrial cell, macrophage, cancer-associated fibroblast, T cells, and mast cells was higher in the high-risk group with *p* value lower than 0.05. The M2 macrophage significantly accumulates in the tumor niche and plays a role in promoting tumor development and immunosuppression, which can be a therapeutic target for treating OC [54–57]. Zhang et al. reported a targeted nanocarrier that could deliver M1-polarizing transcription factors to reprogram TME [58]. Besides, Rodriguez-Garcia et al. reported that folate receptor *β*+ tumor-associated macrophages had the characteristics of macrophages M2, and selective elimination of them by chimeric antigen receptor T cell could retard tumor growth and remodel the TME, which inaugurated a new era in adjuvant therapy of conventional immunotherapy [59]. All these promoted the progress of immune therapy in OC. In recent years, with the development of targeted immune-suppressive therapy, immune checkpoint inhibitors (ICIs) identify specific antigen in the immune cells, help regulate the immune response, and perform the antitumor effect [60–62]. Therefore, we also evaluated the ICIs expression level between two groups, which could future instruct the targeted immune therapy. In conclusion, the results of these studies inspire us to future explore better immunotherapy strategies in OC.

## 5. Conclusion

In summary, by collecting and analyzing the RNA-seq and clinical information of OC samples from TCGA and GTEx database, we identified a new immune-related lncRNA pairing model to predict the prognosis and immune-infiltrating cell condition in human OC, which thus further can instruct immunotherapy. However, a prospective, large-scale, multicenter clinical cohort to validate the prognostic model as well as experimental studies of cell biology to explore the role of related lncRNA in OC is needed.

## Figures and Tables

**Table 1:** The coefficient, HR, 95% confidence interval of HR, and *p* value of each lncRNA pair include in the model.

Id	Coef	HR	HR.95L	HR.95H	*p* value
USP30-AS1|AC008649.2	-0.37265831	0.688900583	0.503499614	0.942570759	0.019821465
AC007389.5|AC073046.1	-0.393910582	0.674414359	0.503652282	0.903072901	0.00818398
AC005884.2|AL163051.1	0.506623749	1.659678235	1.124429293	2.44971548	0.010762253
U62317.1|HOXB-AS2	-0.522539129	0.593012898	0.434220372	0.80987517	0.001015862
BMPR1B-DT|UNC5B-AS1	-0.731071092	0.481393097	0.334014628	0.693799896	0.00000884
AL035701.1|AC106900.1	-0.540417777	0.582504844	0.429107585	0.790738512	0.000528994
NR4A1AS|LINC00893	0.396416384	1.486488139	1.108818372	1.992794349	0.008033751

## Data Availability

The results data used to support the findings of this study are included within the article, and the original data are supplied as supplementary materials in the form of the tables. The software code used to analyze the data are also included within the supplementary materials.

## Abbreviations

OC: Ovarian cancer
TME: Tumor microenvironment
lncRNA: Long noncoding RNA
TCGA: The Cancer Genome Atlas
GTEx: The Genotype-Tissue Expression
DEirlncRNA: Differentially expressed immune-related lncRNA
EMT: Epithelial-mesenchymal transition
HR: Hazard ratio
ROC: Receiver operating characteristic
KM: Kaplan-Meier
AUC: Area under curve
ICIs: Immune checkpoint inhibitors.

## Supplementary Materials

Supplementary 1 Supplementary material 1: 694 immune-related lncRNA and their expression details of each sample.

Supplementary 2 Supplementary material 2: The information of the differentially expressed immune-related lncRNA.

Supplementary 3 Supplementary material 3: The details of 11984 DEirlncRNA pairs in each patient.

Supplementary 4 Supplementary material 4: 227 DEirlncRNA pairs related to overall survival time by univariate Cox analysis.

Supplementary 5 Supplementary material 5: The risk score and the groups of patients.

Supplementary 6 Supplementary material 6: The correlation score of different immune cells in different database.

Supplementary 7 Supplementary material 7: The immune checkpoint genes we analyzed.
