# Supplementary material for: An Immune-Related lncRNA Pairing Model for Predicting the Prognosis and Immune-Infiltrating Cell Condition in Human Ovarian Cancer
Source: Biomed Res Int. 2022 Aug 16;2022:3168408. doi: 10.1155/2022/3168408 (PMC9400430; doi:10.1155/2022/3168408)
Supplement: Supplementary 7 — Supplementary material 7: The immune checkpoint genes we analyzed. [file 3168408.f7.docx]

library(limma)

expFile="symbol.txt"

geneFile="gene.txt"

rt=read.table(expFile, header=T, sep="\t", check.names=F)

rt=as.matrix(rt)

rownames(rt)=rt[,1]

exp=rt[,2:ncol(rt)]

dimnames=list(rownames(exp),colnames(exp))

data=matrix(as.numeric(as.matrix(exp)),nrow=nrow(exp),dimnames=dimnames)

data=avereps(data)

data=data[rowMeans(data)>0,]

gene=read.table(geneFile, header=F, check.names=F, sep="\t")

sameGene=intersect(as.vector(gene[,1]), rownames(data))

geneExp=data[sameGene,]

out=rbind(ID=colnames(geneExp),geneExp)

write.table(out,file="immGeneExp.txt",sep="\t",quote=F,col.names=F)

library(limma)

corFilter=0.4

pvalueFilter=0.001

rt = read.table("lncRNA.txt", header=T, sep="\t", check.names=F)

rt=as.matrix(rt)

rownames(rt)=rt[,1]

exp=rt[,2:ncol(rt)]

dimnames=list(rownames(exp),colnames(exp))

data=matrix(as.numeric(as.matrix(exp)),nrow=nrow(exp),dimnames=dimnames)

data=avereps(data)

data=data[rowMeans(data)>0.5,]

lncRNA=data

rt = read.table("immGeneExp.txt", header=T, sep="\t", check.names=F)

rt=as.matrix(rt)

rownames(rt)=rt[,1]

exp=rt[,2:ncol(rt)]

dimnames=list(rownames(exp),colnames(exp))

immuneGene=matrix(as.numeric(as.matrix(exp)),nrow=nrow(exp),dimnames=dimnames)

immuneGene=avereps(immuneGene)

immuneGene=immuneGene[rowMeans(immuneGene)>0.5,]

outTab=data.frame()

for(i in row.names(lncRNA)){

if(sd(lncRNA[i,])>0.5){

for(j in row.names(immuneGene)){

x=as.numeric(lncRNA[i,])

y=as.numeric(immuneGene[j,])

corT=cor.test(x,y)

cor=corT$estimate

pvalue=corT$p.value

if((cor>corFilter) & (pvalue<pvalueFilter)){

outTab=rbind(outTab,cbind(immuneGene=j,lncRNA=i,cor,pvalue,Regulation="postive"))

}

if((cor< -corFilter) & (pvalue<pvalueFilter)){

outTab=rbind(outTab,cbind(immuneGene=j,lncRNA=i,cor,pvalue,Regulation="negative"))

}

}

}

}

write.table(file="corResult.txt",outTab,sep="\t",quote=F,row.names=F)

immuneLncRNA=unique(as.vector(outTab[,"lncRNA"]))

immuneLncRNAexp=data[immuneLncRNA,]

immuneLncRNAexp=rbind(ID=colnames(immuneLncRNAexp), immuneLncRNAexp)

write.table(immuneLncRNAexp,file="immLncExp.txt",sep="\t",quote=F,col.names=F)

library(limma)

library(pheatmap)

expFile="immlncExp.txt"

fdrFilter=0.01

logFCfilter=2

rt=read.table(expFile, header=T, sep="\t", check.names=F)

rt=as.matrix(rt)

rownames(rt)=rt[,1]

exp=rt[,2:ncol(rt)]

dimnames=list(rownames(exp),colnames(exp))

data=matrix(as.numeric(as.matrix(exp)),nrow=nrow(exp),dimnames=dimnames)

data=avereps(data)

data=data[rowMeans(data)>0,]

conNum=88

treatNum=379

grade=c(rep(1,conNum), rep(2,treatNum))

outTab=data.frame()

for(i in row.names(data)){

geneName=unlist(strsplit(i,"\\|",))[1]

geneName=gsub("\\/", "_", geneName)

rt=rbind(expression=data[i,], grade=grade)

rt=as.matrix(t(rt))

wilcoxTest=wilcox.test(expression ~ grade, data=rt)

conGeneMeans=mean(data[i,1:conNum])

treatGeneMeans=mean(data[i,(conNum+1):ncol(data)])

logFC=log2(treatGeneMeans)-log2(conGeneMeans)

pvalue=wilcoxTest$p.value

conMed=median(data[i,1:conNum])

treatMed=median(data[i,(conNum+1):ncol(data)])

diffMed=treatMed-conMed

if( ((logFC>0) & (diffMed>0)) | ((logFC<0) & (diffMed<0)) ){

outTab=rbind(outTab,cbind(gene=i,conMean=conGeneMeans,treatMean=treatGeneMeans,logFC=logFC,pValue=pvalue))

}

}

pValue=outTab[,"pValue"]

fdr=p.adjust(as.numeric(as.vector(pValue)), method="fdr")

outTab=cbind(outTab, fdr=fdr)

write.table(outTab,file="all.xls",sep="\t",row.names=F,quote=F)

outDiff=outTab[( abs(as.numeric(as.vector(outTab$logFC)))>logFCfilter & as.numeric(as.vector(outTab$fdr))<fdrFilter),]

write.table(outDiff,file="diff.xls",sep="\t",row.names=F,quote=F)

heatmap=rbind(ID=colnames(data[as.vector(outDiff[,1]),]),data[as.vector(outDiff[,1]),])

write.table(heatmap,file="diffLncExp.txt",sep="\t",col.names=F,quote=F)

geneNum=100

outDiff=outDiff[order(as.numeric(as.vector(outDiff$logFC))),]

diffGeneName=as.vector(outDiff[,1])

diffLength=length(diffGeneName)

hmGene=c()

if(diffLength>(2*geneNum)){

hmGene=diffGeneName[c(1:geneNum,(diffLength-geneNum+1):diffLength)]

}else{

hmGene=diffGeneName

}

hmExp=log2(data[hmGene,]+0.01)

Type=c(rep("Normal",conNum),rep("Tumor",treatNum))

names(Type)=colnames(data)

Type=as.data.frame(Type)

pdf(file="heatmap.pdf", height=7, width=10)

pheatmap(hmExp,

annotation=Type,

color = colorRampPalette(c(rep("blue",2), "white", rep("red",2)))(50),

cluster_cols =F,

show_colnames = F,

scale="row",

fontsize = 10,

fontsize_row=2.5,

fontsize_col=10)

dev.off()

pdf(file="vol.pdf",height=5,width=5)

xMax=8

yMax=max(-log10(outTab$fdr))+1

plot(as.numeric(as.vector(outTab$logFC)), -log10(outTab$fdr), xlab="logFC",ylab="-log10(fdr)",

main="Volcano", ylim=c(0,yMax),xlim=c(-xMax,xMax),yaxs="i",pch=20, cex=0.8)

diffSub=subset(outTab, fdr<fdrFilter & as.numeric(as.vector(logFC))>logFCfilter)

points(as.numeric(as.vector(diffSub$logFC)), -log10(diffSub$fdr), pch=20, col="red",cex=1)

diffSub=subset(outTab, fdr<fdrFilter & as.numeric(as.vector(logFC))<(-logFCfilter))

points(as.numeric(as.vector(diffSub$logFC)), -log10(diffSub$fdr), pch=20, col="green",cex=1)

abline(v=0,lty=2,lwd=3)

dev.off()

tcgaPair=data.frame()

rt=read.table("diffLncExp.txt", header=T, sep="\t", check.names=F, row.names=1)

sampleNum=ncol(rt)

for(i in 1:(nrow(rt)-1)){

for(j in (i+1):nrow(rt)){

pair=ifelse(rt[i,]>rt[j,], 1, 0)

pairRatio=sum(pair)/sampleNum

if((pairRatio>0.2) & (pairRatio<0.8)){

rownames(pair)=paste0(rownames(rt)[i],"|",rownames(rt)[j])

tcgaPair=rbind(tcgaPair, pair)

}

}

}

tcgaOut=rbind(ID=colnames(tcgaPair), tcgaPair)

write.table(tcgaOut, file="lncrnaPair.txt", sep="\t", quote=F, col.names=F)

library(limma)

pairFile="lncrnaPair.txt"

cliFile="time.txt"

rt=read.table(pairFile, header=T, sep="\t", check.names=F)

rt=as.matrix(rt)

rownames(rt)=rt[,1]

exp=rt[,2:ncol(rt)]

dimnames=list(rownames(exp), colnames(exp))

data=matrix(as.numeric(as.matrix(exp)), nrow=nrow(exp), dimnames=dimnames)

data=avereps(data)

colnames(data)=gsub("(.*?)\\-(.*?)\\-(.*?)\\-(.*?)\\-.*", "\\1\\-\\2\\-\\3", colnames(data))

data=t(data)

data=avereps(data)

cli=read.table(cliFile,sep="\t",check.names=F,header=T,row.names=1)

sameSample=intersect(row.names(data),row.names(cli))

data=data[sameSample,]

cli=cli[sameSample,]

out=cbind(cli,data)

out=cbind(id=row.names(out),out)

write.table(out,file="pairTime.txt",sep="\t",row.names=F,quote=F)

library(survival)

pFilter=0.01

rt=read.table("pairTime.txt", header=T, sep="\t", check.names=F, row.names=1)

rt$futime=rt$futime/365

outTab=data.frame()

sigGenes=c("futime","fustat")

for(gene in colnames(rt[,3:ncol(rt)])){

cox=coxph(Surv(futime, fustat) ~ rt[,gene], data = rt)

coxSummary = summary(cox)

coxP=coxSummary$coefficients[,"Pr(>|z|)"]

if(coxP<pFilter){

sigGenes=c(sigGenes,gene)

outTab=rbind(outTab,

cbind(gene=gene,

HR=coxSummary$conf.int[,"exp(coef)"],

HR.95L=coxSummary$conf.int[,"lower .95"],

HR.95H=coxSummary$conf.int[,"upper .95"],

pvalue=coxP) )

}

}

write.table(outTab,file="uniCox.txt",sep="\t",row.names=F,quote=F)

surSigExp=rt[,sigGenes]

surSigExp=cbind(id=row.names(surSigExp),surSigExp)

write.table(surSigExp,file="uniSigExp.txt",sep="\t",row.names=F,quote=F)

library(survival)

library(survminer)

library(glmnet)

rt=read.table("uniSigExp.txt", header=T, sep="\t", check.names=F, row.names=1)

x=as.matrix(rt[,c(3:ncol(rt))])

y=data.matrix(Surv(rt$futime,rt$fustat))##

fit <- glmnet(x, y, family = "cox", maxit = 1000)

pdf("lasso.lambda.pdf")

plot(fit, xvar = "lambda", label = TRUE)

dev.off()

cvfit <- cv.glmnet(x, y, family="cox", maxit = 1000)

pdf("lasso.cvfit.pdf")

plot(cvfit)

abline(v=log(c(cvfit$lambda.min,cvfit$lambda.1se)),lty="dashed")

dev.off()

coef <- coef(fit, s=cvfit$lambda.min)

index <- which(coef != 0)

actCoef <- coef[index]

lassoGene=row.names(coef)[index]

lassoGene=c("futime","fustat",lassoGene)

lassoSigExp=rt[,lassoGene]

lassoSigExp=cbind(id=row.names(lassoSigExp),lassoSigExp)

write.table(lassoSigExp,file="lasso.SigExp.txt",sep="\t",row.names=F,quote=F)

rt=read.table("lasso.SigExp.txt",header=T,sep="\t",check.names=F,row.names=1)

multiCox=coxph(Surv(futime, fustat) ~ ., data = rt)

multiCox=step(multiCox, direction="both")

multiCoxSum=summary(multiCox)

outTab=data.frame()

outTab=cbind(

coef=multiCoxSum$coefficients[,"coef"],

HR=multiCoxSum$conf.int[,"exp(coef)"],

HR.95L=multiCoxSum$conf.int[,"lower .95"],

HR.95H=multiCoxSum$conf.int[,"upper .95"],

pvalue=multiCoxSum$coefficients[,"Pr(>|z|)"])

outTab=cbind(id=row.names(outTab),outTab)

outTab=gsub("`","",outTab)

write.table(outTab,file="multi.Cox.txt",sep="\t",row.names=F,quote=F)

riskScore=predict(multiCox, type="risk", newdata=rt)

coxGene=rownames(multiCoxSum$coefficients)

coxGene=gsub("`", "", coxGene)

outCol=c("futime", "fustat", coxGene)

riskOut=cbind(rt[,outCol], riskScore)

riskOut=cbind(id=rownames(riskOut), riskOut)

write.table(riskOut, file="riskScore.txt", sep="\t", quote=F, row.names=F)

bioForest=function(coxFile=null, forestFile=null, forestCol=null){

rt <- read.table(coxFile,header=T,sep="\t",row.names=1,check.names=F)

gene <- rownames(rt)

hr <- sprintf("%.3f",rt$"HR")

hrLow <- sprintf("%.3f",rt$"HR.95L")

hrHigh <- sprintf("%.3f",rt$"HR.95H")

Hazard.ratio <- paste0(hr,"(",hrLow,"-",hrHigh,")")

pVal <- ifelse(rt$pvalue<0.001, "<0.001", sprintf("%.3f", rt$pvalue))

pdf(file=forestFile, width=9, height=7)

n <- nrow(rt)

nRow <- n+1

ylim <- c(1,nRow)

layout(matrix(c(1,2),nc=2),width=c(3,2.5))

xlim = c(0,3)

par(mar=c(4,2.5,2,1))

plot(1,xlim=xlim,ylim=ylim,type="n",axes=F,xlab="",ylab="")

text.cex=0.8

text(0,n:1,gene,adj=0,cex=text.cex)

text(2.08-0.5*0.2,n:1,pVal,adj=1,cex=text.cex);text(2.08-0.5*0.2,n+1,'pvalue',cex=text.cex,font=2,adj=1)

text(3.12,n:1,Hazard.ratio,adj=1,cex=text.cex);text(3.12,n+1,'Hazard ratio',cex=text.cex,font=2,adj=1,)

par(mar=c(4,1,2,1),mgp=c(2,0.5,0))

xlim = c(0,max(as.numeric(hrLow),as.numeric(hrHigh)))

plot(1,xlim=xlim,ylim=ylim,type="n",axes=F,ylab="",xaxs="i",xlab="Hazard ratio")

arrows(as.numeric(hrLow),n:1,as.numeric(hrHigh),n:1,angle=90,code=3,length=0.05,col="darkblue",lwd=2.5)

abline(v=1,col="black",lty=2,lwd=2)

boxcolor = ifelse(as.numeric(hr) > 1, forestCol[1], forestCol[2])

points(as.numeric(hr), n:1, pch = 15, col = boxcolor, cex=1.6)

axis(1)

dev.off()

}

bioForest(coxFile="multi.Cox.txt", forestFile="model.multiForest.pdf", forestCol=c("red","green"))

uniRT=read.table("uniCox.txt",header=T,sep="\t",row.names=1,check.names=F)

uniRT=uniRT[coxGene,]

uniRT=cbind(id=row.names(uniRT), uniRT)

write.table(uniRT, file="unicox.forest.txt", sep="\t", row.names=F, quote=F)

bioForest(coxFile="unicox.forest.txt", forestFile="model.uniForest.pdf", forestCol=c("red","green"))

library(survivalROC) #引用包

rt=read.table("riskScore.txt", header=T, sep="\t", check.names=F, row.names=1)

predictTime=1

roc=survivalROC(Stime=rt$futime, status=rt$fustat, marker=rt$riskScore, predict.time =predictTime, method="KM")

pdf(file="ROC.pdf", width=5.5, height=5.5)

plot(roc$FP, roc$TP, type="l", xlim=c(0,1), ylim=c(0,1),col="black",

xlab="False positive rate", ylab="True positive rate",

lwd = 2, cex.main=1.2, cex.lab=1.2, cex.axis=1.2, font=1.2)

polygon(x=c(0,roc$FP,1,0),y=c(0,roc$TP,1,0),col="#24B35D",border=NA)

text(0.85, 0.1, paste0("AUC=",sprintf("%.3f",roc$AUC)), cex=1.2)

segments(0,0,1,1,lty=2)

dev.off()

predictTime=1

roc=survivalROC(Stime=rt$futime, status=rt$fustat, marker=rt$riskScore, predict.time =predictTime, method="KM")

sum=roc$TP-roc$FP

cutOp=roc$cut.values[which.max(sum)]

cutTP=roc$TP[which.max(sum)]

cutFP=roc$FP[which.max(sum)]

pdf(file="ROC.cutoff.pdf",width=5.5,height=5.5)

plot(roc$FP, roc$TP, type="l", xlim=c(0,1), ylim=c(0,1),col="black",

xlab="False positive rate", ylab="True positive rate",

lwd = 2, cex.main=1.2, cex.lab=1.2, cex.axis=1.2, font=1.2)

polygon(x=c(0,roc$FP,1,0),y=c(0,roc$TP,1,0),col="#24B35D",border=NA)

segments(0,0,1,1,lty=2)

points(cutFP,cutTP, pch=20, col="red",cex=1.5)

text(cutFP+0.15,cutTP-0.05,paste0("Cutoff:",sprintf("%0.3f",cutOp)))

text(0.85, 0.1, paste0("AUC=",sprintf("%.3f",roc$AUC)), cex=1.2)

dev.off()

risk=as.vector(ifelse(rt$riskScore>cutOp,"high","low"))

outTab=cbind(rt, risk)

write.table(cbind(id=rownames(outTab),outTab),file="risk.txt",sep="\t",quote=F,row.names=F)

rocCol=c("red", "green", "blue")

aucText=c()

pdf(file="ROC.multiTime.pdf",width=6,height=6)

predictTime=1

par(oma=c(0.5,1,0,1),font.lab=1.5,font.axis=1.5)

roc=survivalROC(Stime=rt$futime, status=rt$fustat, marker=rt$riskScore, predict.time=predictTime, method="KM")

plot(roc$FP, roc$TP, type="l", xlim=c(0,1), ylim=c(0,1),col=rocCol[1],

xlab="False positive rate", ylab="True positive rate",

lwd = 2, cex.main=1.3, cex.lab=1.2, cex.axis=1.2, font=1.2)

aucText=c(aucText,paste0("one year"," (AUC=",sprintf("%.3f",roc$AUC),")"))

abline(0,1)

predictTime=2

roc=survivalROC(Stime=rt$futime, status=rt$fustat, marker=rt$riskScore, predict.time =predictTime, method="KM")

lines(roc$FP, roc$TP, type="l", xlim=c(0,1), ylim=c(0,1),col=rocCol[2],lwd = 2)

aucText=c(aucText,paste0("two year"," (AUC=",sprintf("%.3f",roc$AUC),")"))

predictTime=3

roc=survivalROC(Stime=rt$futime, status=rt$fustat, marker=rt$riskScore, predict.time =predictTime, method="KM")

lines(roc$FP, roc$TP, type="l", xlim=c(0,1), ylim=c(0,1),col=rocCol[3],lwd = 2)

aucText=c(aucText,paste0("three year"," (AUC=",sprintf("%.3f",roc$AUC),")"))

legend("bottomright", aucText,lwd=2,bty="n",col=rocCol)

dev.off()

library(survival)

library(survminer)

bioSurvival=function(inputFile=null,outFile=null){

rt=read.table(inputFile, header=T, sep="\t")

diff=survdiff(Surv(futime, fustat) ~risk, data = rt)

pValue=1-pchisq(diff$chisq,df=1)

if(pValue<0.001){

pValue="p<0.001"

}else{

pValue=paste0("p=",sprintf("%0.3f",pValue))

}

fit <- survfit(Surv(futime, fustat) ~ risk, data = rt)

surPlot=ggsurvplot(fit,

data=rt,

conf.int=TRUE,

pval=pValue,

pval.size=6,

palette=c("red", "blue"),

legend.title="Risk",

legend.labs=c("High risk", "Low risk"),

xlab="Time(years)",

break.time.by = 1,

risk.table=TRUE,

risk.table.title="",

risk.table.height=.25)

pdf(file=outFile,onefile = FALSE,width = 6.5,height =5.5)

print(surPlot)

dev.off()

}

bioSurvival(inputFile="risk.txt",outFile="survival.pdf")

library(survival) #引用包

risk=read.table("risk.txt", header=T, sep="\t", check.names=F, row.names=1)

cli=read.table("clinical.txt", header=T, sep="\t", check.names=F, row.names=1)

sameSample=intersect(row.names(cli),row.names(risk))

risk=risk[sameSample,]

cli=cli[sameSample,]

rt=cbind(futime=risk[,1],fustat=risk[,2],cli,riskScore=risk[,(ncol(risk)-1)])

uniTab=data.frame()

for(i in colnames(rt[,3:ncol(rt)])){

cox <- coxph(Surv(futime, fustat) ~ rt[,i], data = rt)

coxSummary = summary(cox)

uniTab=rbind(uniTab,

cbind(id=i,

HR=coxSummary$conf.int[,"exp(coef)"],

HR.95L=coxSummary$conf.int[,"lower .95"],

HR.95H=coxSummary$conf.int[,"upper .95"],

pvalue=coxSummary$coefficients[,"Pr(>|z|)"])

)

}

write.table(uniTab,file="uniCox.txt",sep="\t",row.names=F,quote=F)

multiCox=coxph(Surv(futime, fustat) ~ ., data = rt)

multiCoxSum=summary(multiCox)

multiTab=data.frame()

multiTab=cbind(

HR=multiCoxSum$conf.int[,"exp(coef)"],

HR.95L=multiCoxSum$conf.int[,"lower .95"],

HR.95H=multiCoxSum$conf.int[,"upper .95"],

pvalue=multiCoxSum$coefficients[,"Pr(>|z|)"])

multiTab=cbind(id=row.names(multiTab),multiTab)

write.table(multiTab,file="multiCox.txt",sep="\t",row.names=F,quote=F)

bioForest=function(coxFile=null,forestFile=null,forestCol=null){

rt <- read.table(coxFile, header=T, sep="\t", check.names=F, row.names=1)

gene <- rownames(rt)

hr <- sprintf("%.3f",rt$"HR")

hrLow <- sprintf("%.3f",rt$"HR.95L")

hrHigh <- sprintf("%.3f",rt$"HR.95H")

Hazard.ratio <- paste0(hr,"(",hrLow,"-",hrHigh,")")

pVal <- ifelse(rt$pvalue<0.001, "<0.001", sprintf("%.3f", rt$pvalue))

pdf(file=forestFile, width=8, height=5)

n <- nrow(rt)

nRow <- n+1

ylim <- c(1,nRow)

layout(matrix(c(1,2),nc=2),width=c(3,2.5))

xlim = c(0,3)

par(mar=c(4,2.5,2,1))

plot(1,xlim=xlim,ylim=ylim,type="n",axes=F,xlab="",ylab="")

text.cex=0.8

text(0,n:1,gene,adj=0,cex=text.cex)

text(2-0.5*0.2,n:1,pVal,adj=1,cex=text.cex);text(2-0.5*0.2,n+1,'pvalue',cex=text.cex,font=2,adj=1)

text(3.1,n:1,Hazard.ratio,adj=1,cex=text.cex);text(3.1,n+1,'Hazard ratio',cex=text.cex,font=2,adj=1)

par(mar=c(4,1,2,1),mgp=c(2,0.5,0))

xlim = c(0,max(as.numeric(hrLow),as.numeric(hrHigh)))

plot(1,xlim=xlim,ylim=ylim,type="n",axes=F,ylab="",xaxs="i",xlab="Hazard ratio")

arrows(as.numeric(hrLow),n:1,as.numeric(hrHigh),n:1,angle=90,code=3,length=0.05,col="darkblue",lwd=2.5)

abline(v=1,col="black",lty=2,lwd=2)

boxcolor = ifelse(as.numeric(hr) > 1, forestCol, forestCol)

points(as.numeric(hr), n:1, pch = 15, col = boxcolor, cex=1.5)

axis(1)

dev.off()

}

bioForest(coxFile="uniCox.txt", forestFile="uniForest.pdf", forestCol="green")

bioForest(coxFile="multiCox.txt", forestFile="multiForest.pdf", forestCol="red")

library(survivalROC)

riskFile="risk.txt"

cliFile="clinical.txt"

risk=read.table(riskFile, header=T, sep="\t", check.names=F, row.names=1)

risk=risk[,c("futime","fustat","riskScore")]

cli=read.table(cliFile,sep="\t",header=T,check.names=F,row.names=1)

samSample=intersect(row.names(risk), row.names(cli))

risk1=risk[samSample,,drop=F]

cli=cli[samSample,,drop=F]

rt=cbind(risk1, cli)

rocCol=rainbow(ncol(rt)-2)

aucText=c()

pdf(file="cliROC.pdf", width=6, height=6)

par(oma=c(0.5,1,0,1),font.lab=1.5,font.axis=1.5)

roc=survivalROC(Stime=risk$futime, status=risk$fustat, marker=risk$riskScore, predict.time=1, method="KM")

plot(roc$FP, roc$TP, type="l", xlim=c(0,1), ylim=c(0,1),col=rocCol[1],

xlab="False positive rate", ylab="True positive rate",

lwd = 2, cex.main=1.3, cex.lab=1.2, cex.axis=1.2, font=1.2)

aucText=c(aucText,paste0("risk score"," (AUC=",sprintf("%.3f",roc$AUC),")"))

abline(0,1)

j=1

for(i in colnames(rt[,4:ncol(rt)])){

roc=survivalROC(Stime=rt$futime, status=rt$fustat, marker = rt[,i], predict.time =1, method="KM")

j=j+1

lines(roc$FP, roc$TP, type="l", xlim=c(0,1), ylim=c(0,1),col=rocCol[j],lwd = 2)

aucText=c(aucText,paste0(i," (AUC=",sprintf("%.3f",roc$AUC),")"))

}

legend("bottomright", aucText, lwd=2, bty="n", col=rocCol,cex=0.8)

dev.off()

inputFile="risk.txt"

riskScoreFile="riskScore.pdf"

survStatFile="survStat.pdf"

rt=read.table(inputFile, header=T, sep="\t", row.names=1, check.names=F)

rt=rt[order(rt$riskScore),]

riskClass=rt[,"risk"]

lowLength=length(riskClass[riskClass=="low"])

highLength=length(riskClass[riskClass=="high"])

lowMax=max(rt$riskScore[riskClass=="low"])

line=rt[,"riskScore"]

line[line>10]=10

pdf(file=riskScoreFile, width=7, height=4)

plot(line, type="p", pch=20,

xlab="Patients (increasing risk socre)", ylab="Risk score",

col=c(rep("green",lowLength),rep("red",highLength)) )

abline(h=lowMax,v=lowLength,lty=2)

legend("topleft", c("High risk", "Low Risk"),bty="n",pch=19,col=c("red","green"),cex=1.2)

dev.off()

color=as.vector(rt$fustat)

color[color==1]="red"

color[color==0]="green"

pdf(file=survStatFile, width=7, height=4)

plot(rt$futime, pch=19,

xlab="Patients (increasing risk socre)", ylab="Survival time (years)",

col=color)

legend("topleft", c("Dead", "Alive"),bty="n",pch=19,col=c("red","green"),cex=1.2)

abline(v=lowLength,lty=2)

dev.off()

library(limma)

library(ggpubr)

riskFile="risk.txt"

cliFile="clinical.txt"

risk=read.table(riskFile, header=T, sep="\t", check.names=F, row.names=1)

cli=read.table(cliFile, header=T, sep="\t", check.names=F, row.names=1)

samSample=intersect(row.names(risk), row.names(cli))

risk=risk[samSample,"riskScore",drop=F]

cli=cli[samSample,,drop=F]

rt=cbind(risk, cli)

for(clinical in colnames(rt[,2:ncol(rt)])){

data=rt[c("riskScore", clinical)]

colnames(data)=c("riskScore", "clinical")

data=data[(data[,"clinical"]!="unknow"),]

group=levels(factor(data$clinical))

data$clinical=factor(data$clinical, levels=group)

comp=combn(group,2)

my_comparisons=list()

for(i in 1:ncol(comp)){my_comparisons[[i]]<-comp[,i]}

boxplot=ggboxplot(data, x="clinical", y="riskScore", color="clinical",

xlab=clinical,

ylab="Risk score",

legend.title=clinical,

add = "jitter")+

stat_compare_means(comparisons = my_comparisons)

pdf(file=paste0(clinical, ".pdf"), width=6, height=4.5)

print(boxplot)

dev.off()

}

library(ComplexHeatmap)

riskFile="risk.txt"

cliFile="clinical.txt"

risk=read.table(riskFile, header=T, sep="\t", check.names=F, row.names=1)

risk=risk[order(risk$riskScore),]

cli=read.table(cliFile,sep="\t",header=T,check.names=F,row.names=1)

samSample=intersect(row.names(risk), row.names(cli))

risk=risk[samSample,"risk",drop=F]

cli=cli[samSample,,drop=F]

rt=cbind(risk, cli)

sigVec=c("Risk")

for(clinical in colnames(rt[,2:ncol(rt)])){

data=rt[c("risk", clinical)]

colnames(data)=c("riskScore", "clinical")

data=data[(data[,"clinical"]!="unknow"),]

tableStat=table(data)

stat=chisq.test(tableStat)

pvalue=stat$p.value

Sig=ifelse(pvalue<0.001,"***",ifelse(pvalue<0.01,"**",ifelse(pvalue<0.05,"*","")))

sigVec=c(sigVec, paste0(clinical, Sig))

#print(paste(clinical, pvalue, Sig, sep="\t"))

}

colnames(rt)=sigVec

colorList=list(Risk=c("low"="green", "high"="red"))

j=0

for(cli in colnames(rt[,2:ncol(rt)])){

cliLength=length(levels(factor(rt[,cli])))

cliCol=bioCol[(j+1):(j+cliLength)]

j=j+cliLength

names(cliCol)=levels(factor(rt[,cli]))

cliCol["unknow"]="grey75"

colorList[[cli]]=cliCol

}

ha=HeatmapAnnotation(df=rt, col=colorList)

zero_row_mat=matrix(nrow=0, ncol=nrow(rt))

Hm=Heatmap(zero_row_mat, top_annotation=ha)

pdf(file="heatmap.pdf", width=7, height=5)

draw(Hm, merge_legend = TRUE, heatmap_legend_side = "bottom", annotation_legend_side = "bottom")

dev.off()

library(limma)

library(scales)

library(ggplot2)

library(ggtext)

riskFile="risk.txt"

immFile="infiltration_estimation_for_tcga.csv"

risk=read.table(riskFile, header=T, sep="\t", check.names=F, row.names=1)

immune=read.csv(immFile, header=T, sep=",", check.names=F, row.names=1)

immune=as.matrix(immune)

rownames(immune)=gsub("(.*?)\\-(.*?)\\-(.*?)\\-(.*)", "\\1\\-\\2\\-\\3", rownames(immune))

immune=avereps(immune)

sameSample=intersect(row.names(risk), row.names(immune))

risk=risk[sameSample, "riskScore"]

immune=immune[sameSample,]

write.table(file="sameSample.txt", sameSample, sep="\t", quote=F, row.names=F)

x=as.numeric(risk)

outTab=data.frame()

for(i in colnames(immune)){

y=as.numeric(immune[,i])

corT=cor.test(x, y, method="spearman")

cor=corT$estimate

pvalue=corT$p.value

#if(pvalue<0.05){

outTab=rbind(outTab,cbind(immune=i, cor, pvalue))

#}

}

write.table(file="corResult.txt", outTab, sep="\t", quote=F, row.names=F)

corResult=read.table("corResult.txt", head=T, sep="\t")

corResult$Software=sapply(strsplit(corResult[,1],"_"), '[', 2)

corResult$Software=factor(corResult$Software,level=as.character(unique(corResult$Software[rev(order(as.character(corResult$Software)))])))

b=corResult[order(corResult$Software),]

b$immune=factor(b$immune,levels=rev(as.character(b$immune)))

colslabels=rep(hue_pal()(length(levels(b$Software))),table(b$Software))

pdf(file="cor.pdf", width=10, height=15)

ggplot(data=b, aes(x=cor, y=immune, color=Software))+

labs(x="Correlation coefficient",y="Immune cell")+

geom_point(size=3.1)+

theme(panel.background=element_rect(fill="white",size=1,color="black"),

panel.grid=element_line(color="grey75",size=0.5),

axis.ticks = element_line(size=0.5),

axis.text.y = ggtext::element_markdown(colour=rev(colslabels)))

dev.off()

library(limma)

library(ggpubr)

riskFile="risk.txt"

immFile="infiltration_estimation_for_tcga.csv"

risk=read.table(riskFile, header=T, sep="\t", check.names=F, row.names=1)

immune=read.csv(immFile, header=T, sep=",", check.names=F, row.names=1)

immune=as.matrix(immune)

rownames(immune)=gsub("(.*?)\\-(.*?)\\-(.*?)\\-(.*)","\\1\\-\\2\\-\\3",rownames(immune))

immune=avereps(immune)

sameSample=intersect(row.names(risk), row.names(immune))

risk=risk[sameSample, "risk", drop=F]

immune=immune[sameSample,]

data=cbind(risk, immune)

data$risk=factor(data$risk, levels=c("low", "high"))

type=levels(factor(data[,"risk"]))

comp=combn(type, 2)

my_comparisons=list()

for(i in 1:ncol(comp)){my_comparisons[[i]]<-comp[,i]}

for(i in colnames(data)[2:ncol(data)]){

boxplot=ggboxplot(data, x="risk", y=i, fill="risk",

xlab="Risk",

ylab=i,

legend.title="Risk",

palette=c("green", "red")

)+

stat_compare_means(comparisons=my_comparisons)

wilcoxTest=wilcox.test(data[,i] ~ data[,"risk"])

#if(wilcoxTest$p.value<0.05){

j=gsub("/", "-", i)

pdf(file=paste0(wilcoxTest$p.value,j, ".pdf"), width=5, height=4.5)

print(boxplot)

dev.off()

#}

}

library(limma)

library(reshape2)

library(ggplot2)

library(ggpubr)

expFile="symbol-ov.txt"

riskFile="risk.txt"

geneFile="gene.txt"

rt=read.table(expFile, header=T, sep="\t", check.names=F)

rt=as.matrix(rt)

rownames(rt)=rt[,1]

exp=rt[,2:ncol(rt)]

dimnames=list(rownames(exp),colnames(exp))

data=matrix(as.numeric(as.matrix(exp)),nrow=nrow(exp),dimnames=dimnames)

data=avereps(data)

gene=read.table(geneFile, header=F, sep="\t", check.names=F)

sameGene=intersect(row.names(data),as.vector(gene[,1]))

data=t(data[sameGene,])

data=log2(data+1)

ow.names(data)=gsub("(.*?)\\-(.*?)\\-(.*?)\\-(.*?)\\-.*","\\1\\-\\2\\-\\3",row.names(data))

data=avereps(data)

risk=read.table(riskFile, sep="\t", header=T, check.names=F, row.names=1)

sameSample=intersect(row.names(data),row.names(risk))

rt1=cbind(data[sameSample,],risk[sameSample,])

rt1=rt1[,c(sameGene,"risk")]

sigGene=c()

for(i in colnames(rt1)[1:(ncol(rt1)-1)]){

if(sd(rt1[,i])<0.001){next}

wilcoxTest=wilcox.test(rt1[,i] ~ rt1[,"risk"])

pvalue=wilcoxTest$p.value

if(wilcoxTest$p.value<0.05){

sigGene=c(sigGene, i)

}

}

sigGene=c(sigGene, "risk")

rt1=rt1[,sigGene]

rt1=melt(rt1,id.vars=c("risk"))

colnames(rt1)=c("risk","Gene","Expression")

group=levels(factor(rt1$risk))

rt1$risk=factor(rt1$risk, levels=c("low","high"))

comp=combn(group,2)

my_comparisons=list()

for(j in 1:ncol(comp)){my_comparisons[[j]]<-comp[,j]}

boxplot=ggboxplot(rt1, x="Gene", y="Expression", fill="risk",

xlab="",

ylab="Gene expression",

legend.title="Risk",

width=0.8,

palette = c("#0066FF", "#FF0000") )+

rotate_x_text(50)+

stat_compare_means(aes(group=risk),

method="wilcox.test",

symnum.args=list(cutpoints=c(0, 0.001, 0.01, 0.05, 1), symbols=c("***", "**", "*", "ns")), label="p.signif")

pdf(file="checkpoint.diff.pdf", width=8, height=5)

print(boxplot)

dev.off()
